# Effects of Water and Fertilizer Management Practices on Methane Emissions from Paddy Soils: Synthesis and Perspective

**DOI:** 10.3390/ijerph19127324

**Published:** 2022-06-15

**Authors:** Xinyun Gu, Shimei Weng, Yu’e Li, Xiaoqi Zhou

**Affiliations:** 1 Zhejiang Tiantong Forest Ecosystem National Observation and Research Station, Center for Global Change and Ecological Forecasting, School of Ecological and Environmental Sciences, East China Normal University, Shanghai 200241, China; g.x.y111@hotmail.com (X.G.); shmweng@163.com (S.W.); 2 Institute of Environment and Sustainable Development in Agriculture, Chinese Academy of Agricultural Sciences, Beijing 100081, China; liyue@caas.cn

**Keywords:** water management practices, fertilizer management practice, CH_4_ emissions, CH_4_ oxidation, CH_4_ production, paddy field, meta-analysis

## Abstract

Water and fertilizer management practices are considered to have great influence on soil methane (CH_4_) emissions from paddy fields. However, few studies have conducted a quantitative analysis of the effects of these management practices. Here, we selected 156 observations of water management from 34 articles and 288 observations of fertilizer management from 37 articles and conducted a global meta-analysis of the effects of water and fertilizer management practices on soil CH_4_ emissions in paddy fields. In general, compared with traditional irrigation (long-term flooding irrigation), water-saving irrigation significantly decreased soil CH_4_ emissions but increased rice yield. Among the different practices, intermittent irrigation had the fewest reductions in CH_4_ emissions but the greatest increase in rice yield. In addition, fertilization management practices such as manure, mixed fertilizer (mixture), and straw significantly enhanced CH_4_ emissions. Rice yields were increased under fertilization with a mixture, traditional fertilizer, and controlled release fertilizer. Our results highlight that suitable agricultural water and fertilizer management practices are needed to effectively reduce CH_4_ emissions while maintaining rice yields. We also put forward some prospects for mitigating soil CH_4_ emissions from paddy fields in the context of global warming in the future.

## 1. Introduction

Methane (CH_4_) is the second-largest greenhouse gas after carbon dioxide (CO_2_) in the atmosphere. Since the pre-Industrial era, atmospheric CH_4_ concentrations have rapidly increased from 700 to 1803 parts per billion [1]. On a molecular scale, the global warming potential of CH_4_ is around 25 times than that of CO_2_, and contributes ~25% of global warming [1]. Given that small changes in atmospheric CH_4_ concentrations can have a strong impact on global warming [2], the mitigation of CH_4_ emissions, particularly in paddy fields, has received wide attention.

Paddy fields are one of the most important sources of anthropogenic CH_4_ emissions [3,4]. The global CH_4_ emissions from paddy fields are 31–280 Tg yr^−1^, accounting for 10–20% of anthropogenic CH_4_ emissions [5]. Most of the CH_4_ emissions from paddy fields are concentrated in parts of the tropical, subtropical, and northern temperate zones, such as Central America, Latin America, Africa, and Southeast Asia [6]. Among these, Southeast Asia accounted for ~90% of the global CH_4_ emissions from paddy fields, whereas global CH_4_ emissions from Africa and South America have increased by 3.5% and 4.7%, respectively [7]. Most rice cultivation is concentrated in latitudes from 50°N to 30°S [6]. The spatial distribution of paddy fields corresponds to global CH_4_ emissions, indicating the importance of paddy fields to global CH_4_ emissions [7].

With the continuous growth in the global population, modeling predictions have shown that the rice yield in the future needs to increase at the rate of 8–10 × 10^9^ kg yr^−1^ in order to meet the basic needs of human development [8]. The continuous cultivation of rice planting will, in turn, lead to a gradual increase in atmospheric CH_4_ concentrations [9]. In addition, it is worth noting that CH_4_ emissions from paddy fields may continue to increase under global warming and changes in rainfall patterns in the future [10]. Therefore, it is a necessary to develop suitable agricultural practices that can not only increase production but also reduce CH_4_ emissions [11].

**Figure 1:**
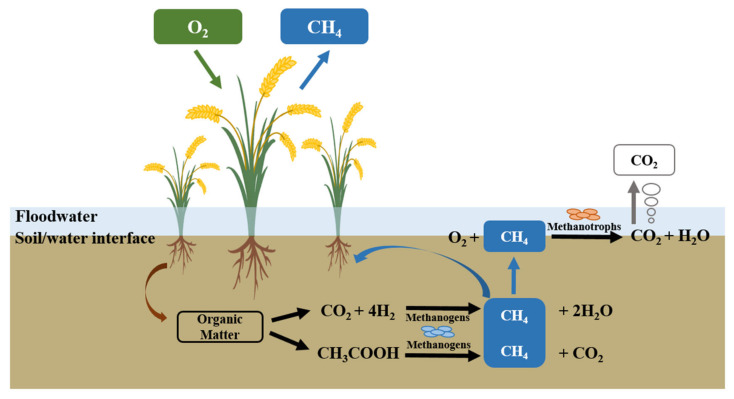
Schematic graph showing the three processes of CH_4_ in paddy soils: CH_4_ is first produced in the anaerobic environment by methanogens, then transported to the soil–water interface and the rice rhizosphere, and can then be oxidized by methanotrophs in an aerobic environment before being released into the atmosphere.

CH_4_ emissions from paddy fields are a comprehensive result of the three processes of CH_4_ production, oxidation, and transportation to the atmosphere, as shown in Figure 1 [12]. Soil CH_4_ production is a process by which organic matter is hydrolyzed, fermented, and converted into CH_4_ by methanogens and other microorganisms under anaerobic conditions [13]. About 30–90% of CH_4_ is oxidized by methanotrophs in the soil’s aerobic zone (i.e., the topsoil and rice rhizosphere) before it is released into the atmosphere [14,15], and about 15–30% of CH_4_ is released into the atmosphere through plants and the soil surface [16]. There are many factors that influence soil CH_4_ production and oxidation processes in paddy fields, such as the soil moisture content, pore oxygen content, and the carbon and nitrogen contents [17,18,19]. These environmental factors mainly affect soil CH_4_ emissions by influencing the community structure and activity of methanogens and methanotrophs in paddy fields.

At present, it is clear that different management practices such as irrigation and fertilization have a strong impact on soil CH_4_ production and oxidation processes, and thus affect CH_4_ emissions in paddy fields. However, few studies have reported the effects of different water and fertilizer management practice regimes on CH_4_ emissions from paddy soils comprehensively. Here, we collected data on the effects of water and fertilizer managements on soil CH_4_ emissions in paddy fields from 1990 to 2019 and conducted a meta-analysis. The objectives of this study were to (1) quantify the effects of different water and fertilizer management practices on CH_4_ emissions from paddy fields, and (2) put forward environmentally friendly water and fertilizer management practices that can increase yield and also decrease CH_4_ emissions.

## 2. Materials and Methods

### 2.1. Effects of Water Management on Soil CH_4_ Emissions and Rice Yield

**Figure 2:**
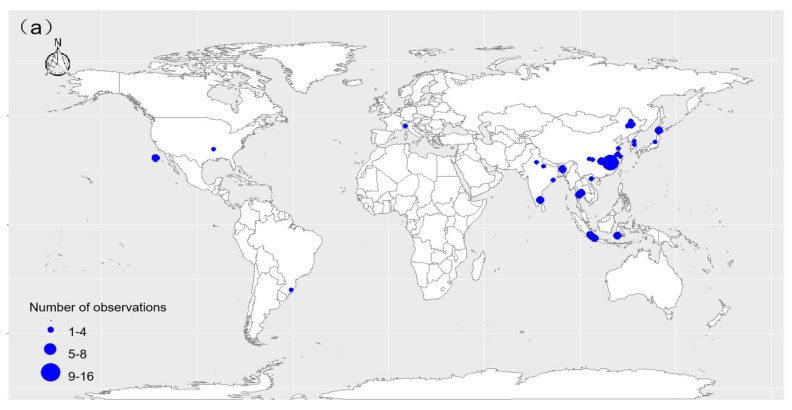

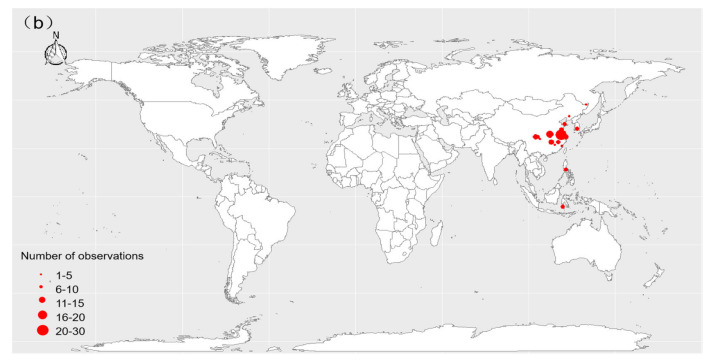
The distribution of the study plots of water (**a**) and fertilizer (**b**) management practices in paddy fields around the world. The size of the dots indicates the number of data observations.

Peer-reviewed journal articles were systematically searched in the Web of Science database with the search term combinations [water managements OR irrigation] AND [CH_4_ emission OR CH_4_] AND [rice OR paddy field]. All the articles revealed through the search were reviewed and those that met the following three criteria were selected: (1) the study conducted in situ CH_4_ flux measurements for at least one growing season; (2) soil CH_4_ flux could be extracted from the text, tables, and figures directly, or could be calculated from cumulative soil CH_4_ emissions and rice growth period; (3) irrigation and other field management practices were directly given in the articles. When one publication contained several experiments under different conditions, such as different locations, rice varieties, or fertilizer managements, we considered these as different observations. In total, 156 datasets for soil CH_4_ emissions from paddy fields from 37 sites in 36 articles on water management practices were found (Figure 2a, Table S1). Six common water-saving irrigation methods were collected through the data collection process: flooding–drainage–flooding–moist (FDFM), intermittent irrigation (II), rainfed (RF), flooding–drainage–flooding (FDF), alternating wet and dry (AWD), and moist irrigation (MI). All of the studies used automated or manual static chamber methods. For each selected study, we collected data on the latitude, longitude, climate zone, water management, fertilizer management, and rice yield. GetData was used to digitally extract the data from figures when the results were reported graphically. Traditional irrigation (long-term flooding irrigation) and treatment means, standard deviations, and sample sizes (*n*) of CH_4_ flux and rice yield were directly extracted or recalculated; CH_4_ emission rates and rice yield were standardized to mg (CH_4_) m^−2^ h^−1^ and kg hm^−2^, respectively.

### 2.2. Effects of Fertilizer Management on Soil CH_4_ Emissions and Rice Yield

In a similar method to that for water management, peer-reviewed journal articles were systematically searched in the Web of Science database, with the search term combinations [fertilizer managements OR fertilization OR nitrogen] AND [CH_4_ emission OR CH_4_] AND [rice OR paddy field]. Articles were selected according to the following criteria: (1) the study conducted in situ soil CH_4_ flux measurements for one growing season at least; (2) soil CH_4_ flux could be extracted from the text, tables, and figures directly, or could be calculated from the cumulative soil CH_4_ emissions and the rice growth period; (3) the study clearly described the fertilization practice used. When one publication applied multiple fertilization and water management regimes, different data under the same water management conditions were chosen and considered to be different observations. In 34 fertilizer management papers, we collected 288 soil CH_4_ flux datasets from 37 sites around the world (Figure 2b, Table S2). In this study, six different fertilizer management practices were found: controlled release fertilizer (CRF), straw (S), biochar (BC), mixed fertilizer (Mix), manure (M), and traditional fertilizer (TF). Straw and biochar were included for simplicity, even if they are not fertilizers, but soil improvers/amendments. For each selected study, our information collection standards and data processing methods were consistent with those described in the water management section (see *Effects of Water Managements on Soil CH_4_ Emissions and Rice Yield* for details). The effects of fertilizer management treatments were compared with no fertilizer plots.

### 2.3. Statistical Analysis

The data were analyzed to quantify the impact of different water and fertilizer management practices on the soil CH_4_ emissions from paddy fields. Defined as the “effect size”, the natural log of the response ratio was used to assess the responses of soil CH_4_ emissions to the treatments. We calculated the response ratios from each study via the methods described by Zhou et al. [20] and Feng et al. [21]. Briefly, the effect size was calculated as follows:

ln (*X_i_/X_n_*) = ln *X_i_* − ln *X_n_* (1)

where *X_i_* and *X_n_* are the values of each observation in the treatment and the corresponding traditional irrigation (long-term flooding irrigation) or no fertilizer plots, respectively. The sampling variance for each effect size was calculated as:

ln [(1/*n_i_*) × (*S_i_*/*X_i_*)^2^ + (1/*n_n_*) × (*S_n_*/*X_n_*)^2^] (2)

where *n_i_* and *n_n_*, *S_i_* and *S_n_*, and *X_i_* and *X_n_* are the treatments’ and traditional irrigation (long-term flooding irrigation) or no fertilizer’s sample sizes, standard deviations, and mean responses, respectively. The effects on soil CH_4_ emissions from paddy fields and the differences between the treatment and traditional irrigation (long-term flooding irrigation) or no fertilizer plots were considered to be significant if the 95% confidence interval of effect size did not overlap zero.

## 3. Results and Discussions

### 3.1. Impact of Water Management on CH_4_ Emissions from Paddy Fields and Suitable Management Recommendations

**Figure 3:**
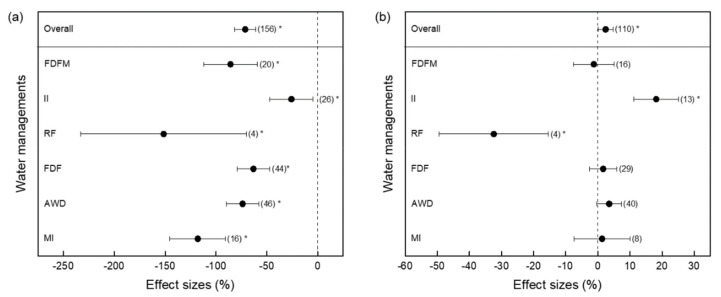
Effects of different water management practices on CH_4_ efflux (**a**) and rice yield (**b**) in paddy fields. * Indicates significant levels. Numbers in parentheses show the number of data observations. Vertical dotted lines at 0% indicate the effect of the control. FDFM, flooding–drainage–reflooding–moist; II, intermittent irrigation; RF, rainfall; FDF, flooding–drainage–reflooding; AWD, alternating wet and dry; MI, moist irrigation. Overall, all the treatments.

Suitable water management practices are considered to be one of the most effective measures for decreasing soil CH_4_ emissions from paddy fields. In general, compared with traditional irrigation (long-term flood irrigation), water-saving irrigation significantly decreased soil CH_4_ emissions by 71.25% (Figure 3a). Among these practices, RF had the greatest reductions, with a reduction rate of 151.45%, followed by MI with a reduction rate of 117.88%. However, the emission reduction rate of II was the lowest: only 25.78% (as shown in Figure 3a). Suitable irrigation must supply the water requirements during rice growth and development, which will not only decrease CH_4_ emissions but will also ensure rice yield [22]. According to all the rice yield data from several global water management practices, water-saving irrigation significantly increased rice yield by 2.4%. Among these practices, II management had the greatest increase in rice yield (18.15%), whereas RF irrigation markedly reduced rice yield by 32.40% as the water did not meet the needs of rice growth. Other irrigation practices had no effect on rice yield (Figure 3b). It is worth noting that the RF treatment had the greatest variance because it had fewer observations, which may overstate the effect of this treatment.

Previous studies have shown that water-saving irrigation can promote crops’ root activity, improve soil fertility, and reduce soil pore water content, thus introducing oxygen (O_2_) into the soil [23]. Given that methanogens are anaerobic microorganisms, more oxygen gas can inhibit the activity of methanogens but can increase the activity of methanotrophs, thus ultimately reducing soil CH_4_ emissions [23,24]. In addition, appropriate drainage measures can promote nitrogen absorption by plants and improve soil oxidation conditions [9]. Once the water table has been restored, rice can recover quickly from relatively short-term water stress. In most parts of Southeast Asia, this measure has been shown to decrease CH_4_ emissions while maintaining rice yield. Studies have shown that rice is more tolerant to water stress before flowering, whereas the lowering and heading periods are more sensitive to mid-drainage [16]. Therefore, it is necessary to consider the water demand patterns of rice at different stages and carry out reasonable irrigation.

Concurrent with global warming, the rainfall patterns are likely to change greatly by the end of this century, which will markedly influence CH_4_ emissions from paddy fields [10]. Therefore, it is necessary to explore appropriate water management practices to mitigate CH_4_ emissions from paddy fields in the context of global warming. Previous studies have shown that, compared with continuous flooding irrigation, water-saving irrigation effectively decreased CH_4_ emissions in the growing season by improving soil aeration and increasing soil permeability [23,24,25]. In addition, according to previous studies, rice yield displays dynamic changes under different soil moisture conditions. When the soil moisture content is as low as 60% of the saturated volumetric water content, rice has the risk of reduced yield [26]. Therefore, in future agricultural management practices, we should choose water-saving measures such as thin and shallow water-saving irrigation in which the lower limit of irrigation at the early tillering stage and milk stage is within the “safety threshold” range, thus decreasing CH_4_ emissions while maintaining the rice yield.

Irrespective of whether conventional irrigation or water-saving irrigation was used, CH_4_ emissions during the growth season was concentrated in the stage from green-up to tillering, which accounted for about 85% of the total CH_4_ emissions in the growth season. This indicated that the peak of CH_4_ flux is mainly concentrated in the vegetative growth stage of rice, and the mid-drainage treatment reduced soil CH_4_ emissions in the late growth stage. Even if the paddy is re-flooded after the mid-drainage treatment, soil CH_4_ flux still remains at a low level unlike that before the mid-drainage [27]. In addition, during the drainage period, there is no water layer on the soil surface and the soil permeability is maximized, so the O_2_ in the air rapidly diffuses to the soil and the soil Eh increases sharply, which then improves the soil’s CH_4_ oxidation capacity, resulting in a rapid reduction in the CH_4_ emissions flux from the paddy field [19,28]. After re-flooding, rice enters the reproductive growth stage. Rice at this stage is highly sensitive to water and requires frequent irrigation, which introduces sufficient O_2_ into the soil and takes away part of the dissolved CH_4_ and organic substrate, thus further reducing CH_4_ flux [19]. Therefore, one effective measure to decrease CH_4_ emissions is to clarify the water demand of rice in the key period of growth and development and then irrigate on demand.

Compared with conventional irrigation measures, thin and shallow water-saving irrigation effectively inhibited soil CH_4_ emissions, which can largely be attributed to the lower CH_4_ flux during the tillering stage [29]. Compared with traditional irrigation, paddy fields under water-saving irrigation are subjected to drying and rewetting, and the soil permeability is improved, so it is subjected to both aerobic and anaerobic environments [30,31]. This can promote soil CH_4_ oxidation and production, which can decrease soil CH_4_ emissions [32]. Previous studies have also reported that early drying of fields can effectively inhibit the CH_4_ flux during the vegetative growth stage of rice [27,33,34]. However, at the beginning of mid-drainage and the end of drying until maturity, paddy soils under water-saving irrigation remain in a state of drying and rewetting, which further reduces soil CH_4_ emissions. According to the comprehensive results of the meta-analysis, MI produced the best reductions in CH_4_ emissions while maintaining rice yield. Although the reductions in CH_4_ emission of II were lower than those of MI, it had a stronger effect on increasing rice yield (Figure 3). As a whole, during the process of rice growth, a suitable irrigation measure can be selected according to the actual situation.

### 3.2. Impact of Fertilizer Management on CH_4_ Emissions in Paddy Fields and Suitable Management Recommendations

**Figure 4:**
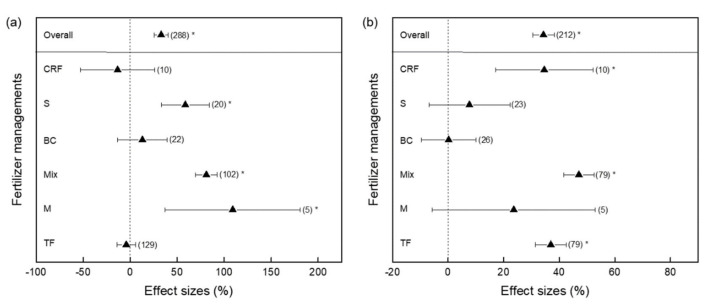
Effects of different fertilizer management practices on CH_4_ efflux (**a**) and rice yield (**b**) in paddy fields. * Indicates significant levels. Numbers in parentheses show the number of data observations. Vertical dotted lines at 0% indicate the effect of the control. CRF, controlled release fertilizer; S, straw; BC, biochar; Mix, mixture; M, manure; TF, traditional fertilizer. Overall, all the treatments.

Fertilizer is one of the important factors affecting soil CH_4_ emissions from paddy fields. In general, compared with no fertilizer, the addition of fertilizer significantly promoted soil CH_4_ emissions, with an increase of 32.98%. Among the different practices, M, S, and Mix significantly increased CH_4_ emissions (Figure 4a). However, there were no responses of CH_4_ emissions to CRF, BC, and TF (Figure 4a). In addition, we should take yield into account when reducing soil CH_4_ emissions. In summary, according to the 212 datasets, fertilization significantly increased rice yield by 34.3%. Mixed fertilization increased rice yield by 47.0%, followed by TF and CRF, which increased rice yield by 36.96% and 34.61%, respectively. However, S, BC, and M had no effect on rice yield (Figure 4b). As mentioned before, the higher variance of the M treatment also needs to be noticed, as it may not represent the effect in all the plots under the M treatment.

The ability of fertilization to promote soil CH_4_ emissions might be attributed to (1) the increase in substrate sources for methanogens, or (2) the decrease in soil redox potential, which provides favorable environmental conditions for the growth of methanogens, thus increasing soil CH_4_ emissions [35]. However, different fertilizer types have different effects on soil CH_4_ emissions, which might be caused by different quality and quantity of substrates for methanogens [36]. Compared with a single application of chemical fertilizer, the mixed treatment had higher soil C/N, thus increasing the abundance of soil methanogens and effectively increasing soil CH_4_ production [29]. Therefore, to mitigate soil CH_4_ emissions from paddy fields, we should consider not only the impact of different fertilizer types, but also reasonable application schemes based on the local situation.

Fertilization types applied in paddy fields mainly include organic fertilizers and chemical fertilizers. Organic fertilizers include straw, manure, and biogas residue, whereas inorganic fertilizers include nitrogen, potassium, and phosphorus. Our meta-analysis showed that fertilization significantly increased CH_4_ emissions from paddy soils (Figure 4). The total CH_4_ emissions of treatments returning rice straw are significantly higher than those that did not return straw across the entire growth period of rice [37]. Compared with straw-returning treatments, burning straw in-situ can significantly decrease soil CH_4_ emissions [37]. On the other hand, the effects of straw application timing on soil CH_4_ emissions cannot be ignored. The contribution of straw application before winter to the greenhouse effect is less than that before rice transplanting [38]. Compared with applying straw before the winter cropping season, applying straw before transplanting rice significantly increases soil CH_4_ emissions from paddy fields [38]. Given that returning straw directly increases soil CH_4_ emissions from paddy fields, the straw returning technique should be reduced as far as possible. The recommendation for this measure is that straw can be applied before the winter cropping season to decrease soil CH_4_ emissions from paddy fields.

The use of agricultural fertilizers, such as animal manure, can effectively improve rice yields, but many studies have indicated that its use can increase soil CH_4_ emissions [39,40]. For example, some studies have shown that the application of pig manure and biogas residue can increase soil CH_4_ emissions from paddy fields. However, the application of decomposed bacterial residue and biogas residue instead of fresh straw and cow manure can significantly decrease soil CH_4_ emissions from paddy fields without influencing rice yield [40]. Given that the effect of manure fermentation or composting is better than that of direct application, in order to maintain or increase soil fertility and rice yield, our recommendation for reducing soil CH_4_ emissions from paddy fields is to use fermented manure instead of untreated manure and straw.

Regarding the effects of fertilizer on CH_4_ emissions from paddy fields, the conclusions of each study are different (Figure 4). Studies have shown that CH_4_ emissions are affected by CH_4_ production and oxidation potential, and that the ammonia nitrogen content of different soils is different. Further studies need to investigate underlying microbial mechanisms [23,41].

Previous studies have shown that improved nitrogen fertilizer can influence soil CH_4_ emissions from paddy fields [42]. The purpose of using improved nitrogen fertilizer is to regulate the process of nitrogen transformation and reduce nitrogen losses, thus altering the soil-available nitrogen, influencing rice plants and the soil microbial community, and ultimately affecting CH_4_ emissions from paddy fields [42,43]. It has been reported that, compared with conventional nitrogen fertilizers such as urea, the application of CRF during rice growth can significantly decrease CH_4_ emissions from paddy fields [29,44]. Some studies have also reported that the use of CRF can markedly increase grain yield [44]. The reason for this is that the nitrogen of CRF is released over several months, and the nitrogen content in the soil is relatively balanced throughout the entire rice growth period, thus maintaining soil CH_4_ oxidation capacity but inhibiting soil CH_4_ emissions [45]. Considering both soil CH_4_ emissions and rice yield, we recommend CRF as the most cost-effective method of field fertilization, which has a tendency to decrease CH_4_ emissions from paddy fields.

At present, there have been many studies on the effects of different water and fertilizer management practices on soil CH_4_ emissions from paddy fields [12,23,41,46], but only a few studies are available on their interactive effects on soil CH_4_ emissions. Most of them focused on the interaction between traditional nitrogen fertilizer and intermittent irrigation [29], but few studies are available on the interactions of MI and improved fertilizer and their effects on soil CH_4_ emissions. Therefore, our suggestion is that future research can focus on this issue as the key object, as these measures may be a win–win field management strategy for lower soil CH_4_ emissions and higher rice yield.

## 4. Conclusions and Prospects

Mitigation of soil CH_4_ emissions in paddy fields has become a hot issue not only in climate research but also in the fields of environment and agriculture. In the past decade, a large number of in situ studies on CH_4_ emissions in paddy fields have been conducted [47,48,49], and the focus of future studies should be to build a comprehensive model of soil CH_4_ emissions based on the existing data and to predict the changes in soil CH_4_ emissions from paddy fields. In addition, the differences in soil CH_4_ emissions from different regions and environments are also worth investigating. The microbial mechanisms of CH_4_ production and emissions from paddy fields needs more investigations, which will help us adopt appropriate agronomic measures.

### 4.1. Regulatory Effects of Rice Varieties on Soil CH_4_ Emissions

The selected rice variety can have an impact on soil CH_4_ emissions from paddy fields [28,50]. Studies have confirmed that rice plants are one of the pathways of CH_4_ transportation from soils to the atmosphere through its well-developed aerenchyma [16]. Therefore, soil CH_4_ produced by methanogens enters the root system of rice plants and is transported through the aerenchyma by diffusion, and is then released into the atmosphere (Figure 1). Therefore, an important mitigation measure for decreasing soil CH_4_ emissions is to cultivate rice varieties with poor aeration tissue to inhibit the CH_4_ transport pathway [51]. In particular, rice varieties affect soil CH_4_ emissions because rice plants mediate the transportation of CH_4_ and O_2_ through the aerenchyma [52] and provide substrates for methanogens and methanotrophs through root exudates [53]. Therefore, differences between rice cultivars may play a major role in the regulation of soil CH_4_ emissions from paddy fields.

Previous studies showed that the CH_4_ emission of super rice varieties were lower than those of conventional rice and hybrid rice, and that the soil CH_4_ emissions of hybrid rice were lower than those of conventional rice. The reason for these results might be because high-yielding rice varieties lock more substrates into the rice plants, thus decreasing soil CH_4_ emissions [28,54]. It has also been reported that the cultivation of early-maturing varieties can effectively reduce CH_4_ emissions from paddy soils, and some studies have reported that the cultivation of early maturing varieties significantly changed the abundance of methanogens and methanotrophs in soil, thus affecting CH_4_ fluxes [50]. Therefore, to consider the social economic and environmental benefits comprehensively, super rice and hybrid rice can be used when sowing, which can not only decrease soil CH_4_ emissions from paddy fields but also increase rice yield. In addition, selecting early-maturing varieties is also an effective planting strategy in certain regions.

### 4.2. Influence of Soil Food Webs on Soil CH_4_ Emissions from Paddy Fields

The soil CH_4_ flux is the combined result of the processes of CH_4_ production and oxidation, which are mediated by methanogens and methanotrophs, respectively [13]. Therefore, soil CH_4_ production can be inhibited by decreasing the activity and abundance of methanogens, whereas soil CH_4_ oxidation can be promoted by increasing the activity and abundance of methanotrophs, thus decreasing soil CH_4_ emissions [13,55,56]. At present, many studies have identified the key environmental driving factors controlling the activities of methanogens and methanotrophs, such as physical and chemical factors, temperature, nitrogen, and metal content [57,58,59]. However, methanogens and methanotrophs have complex dynamic relationships with environmental factors and with complex biological components, such as the structure of the nutrient food webs of microbial networks, but these factors have rarely been studied [60].

Soil methanogens and methanotrophs are important for transferring energy and biomass to higher nutritional levels [61]. It has been reported that some bacteria can obtain the nitrogen sources they need to grow and metabolize by infecting methanogens, and some anaerobic ciliates can obtain nutrients from methanogens to satisfy their own growth [62]. The activities of these soil organisms greatly affect the soil methanogenic community, thus affecting soil CH_4_ fluxes. In addition to methanogens, methanotrophs act as natural filters through soil CH_4_ consumption [63], which can decrease the amount of CH_4_ released into the atmosphere by approximately 90% [64].

It has been suggested that soil organisms, such as amoeba and flagellates, can feed on methanogens and methanotrophs, and predation pressure regulates the community structure of soil methanogens and methanotrophs [62,65]; however, relevant studies have rarely reported on the rice field ecosystem. Therefore, we recommend using the model of predator–prey relationships and micro-predators in the soil microbial community to quantify whether predation or grazing is an important biological factor affecting CH_4_ consumption.

Quantifying the effects of water and fertilizer practices on soil CH_4_ emissions in paddy fields is important for mitigating global warming in the future. Our synthesis, based on an extensive database, showed that water-saving irrigation significantly decreased soil CH_4_ emissions but significantly increased rice yield. In addition, fertilization significantly enhanced CH_4_ emissions and rice yields. To achieve a win–win field management strategy for lowering soil CH_4_ emissions and increasing rice yields, studies need to focus on the interactions among fertilizers, intermittent irrigation, and the associated underlying microbial mechanisms, thus building comprehensive models to improve our understanding and achieving sustainable development in agricultural ecosystems.

## Data Availability

All data presented here are contained in previously published literature.

## Supplementary Materials

The following supporting information can be downloaded at: https://www.mdpi.com/article/10.3390/ijerph19127324/s1, Table S1. The location of the study plots of water management practices and the soil classification in paddy fields; Table S2. The location of the study plots of fertilizer management practices and the soil classification in paddy fields.
