# Supplementary material for: Effects of Water and Fertilizer Management Practices on Methane Emissions from Paddy Soils: Synthesis and Perspective
Source: Int J Environ Res Public Health. 2022 Jun 15;19(12):7324. doi: 10.3390/ijerph19127324 (PMC9223590; doi:10.3390/ijerph19127324)
Supplement: Supplementary file 1 [file ijerph-19-07324-s001.zip › ijerph-1656743-supplementary.pdf]

## Supplementary Information

**Table S1.** The location of the study plots of water management practices and the soil classification in paddy fields.

| Country | Location | Soil classification | Reference |
|---|---|---|---|
| China | 34°63′ N, 121°05′ E | Hydragric Anthrosol | [1] |
| Thailand | 14°21′ N, 100°36′ E | Ayutthaya soil series | [2] |
| China | 28°11′ N, 113°04′ E | Endogleyic Hapli-Stagnic Anthrosol | [3] |
| China | 30°05′ N, 104°34′ E | purple shale | [4] |
| South Korea | 38°17′ N, 128°7′ E | Terric cambisols or even as anthrosols | [5] |
| South Korea | 36°50′ N, 128°26′ E | Typic Haplaquents with somewhat impeded drainage | [6] |
| Japan | 43°01′N, 141°25′E | Allophanic Andosols | [7] |
| China | 32°30′ N, 119°25′ E | sandy loam | [8] |
| China | 30°28′ N, 114°21′ E | silt loam | [9] |
| Brazil | 29°55'S, 50°58′ W | Entisol | [10] |
| Japan | 37°54′ N, 139°3′ E | Fluvaquents | [10] |
| China | 31°15′ N, 120°57′ E | Hydragric Anthrosol | [11] |
| China | 31°52′ N, 118°50′ E | Gleysols or hydromorphic | [12] |
| USA | 34°35′ N, 91°45′ W | Clay in the north field and silty clay or silty clay loam in the south field | [13] |
| Bangladesh | 24°75′ N, 90°50′ E | silt loam | [14] |
| China | 28°20′ N, 116°5′ E | Stagnic Anthrosols | [15] |
| China | 28°55′ N, 111°30′ E | Stagnic Anthrosols | [16] |
| India | 26°45′ N, 80°51′ E | sandy loam | [17] |
| China | 30°27′ N, 121°43′ W | EsquonNeerdobe | [18] |
| Italy | western area of the plain of the river Po | Fluvaquentic Epiaquept | [19] |
| China | 31°52′ N, 118°50′ E | Hydromorphic soil | [20] |
| China | 28°55′ N, 111°27′ E | Stagnic Anthrosols | [21] |
| China | 29°48′ N, 106°24′ E | Cabhaplic Stagnic Anthrosols | [22] |
| China | 45°00′ N, 124°43′ E | Saline–alkaline paddy soil | [23] |
| Indonesia | 4°59'S, 119°34′ E | Typic Haplusterts | [24] |

| | | | |
|---|---|---|---|
| Thailand | 13°35′ N, 99°30′ E | Alfisols | [25] |
| China | 28°37′ E, 112°80′ N | Stagnic Anthrosol | [26] |
| China | 47°26′ N, 126°38′ E | black soil | [27] |
| China | 31°52′ N, 118°50′ E | silt loam | [28] |
| India | 28°40′ N, 77°12′ E | Typic Ustochrept and loam | [29] |
| India | 11°0′ N, 79°30′ E | Alluvial clay | [30] |
| Vietnam | 20°55′ N, 105°50′ E | soft soils, kaolinite and chlorite clay minerals | [31] |
| China | 45°49′ N, 126°48′ E | chernozem soil | [32] |
| Indonesia | Taman Bogo, Central Lampung, Southern Sumatra | Typic Paleudult (Red Yellow Podzolic Soil) | [33] |
| Indonesia | 6°20'S, 107°39′ E | Aerie Tropaqualf | [34] |
| India | 20°25′ N, 85°55′ E | Haplaquept | [35] |

**Table S2.** The location of the study plots of fertilizer management practices and the soil classification in paddy fields.

| **Country** | **Location** | **Soil classification** | **reference** |
|---|---|---|---|
| China | 32°35′ N, 119°42′ E | Anthrosols | [36] |
| China | 31°12′ N, 121°08′ E | Anthrosols | [37] |
| China | 31°52′ N, 118°50′ E | Irragric Anthrosols | [38] |
| China | 31°03′ N, 104°10′ E | Hydragric Anthrosol | [39] |
| China | 28°15′ N, 116°55′ E | Stagnic Anthrosol | [40] |
| China | 32°10′ N, 112°10′ E | unknow | [41] |
| South Korean | 35°8′ N, 128°5′ E | Pyeongtaeg Series | [42] |
| South Korean | 35°8′ N, 128°5′ E | Pyeongtaeg series | [43] |
| China | 31°24′ N, 119°41′ E | hydroagric Stagnic Anthrosol and entic Halpudept | [44] |
| Japan | 14°16′ N, 121°26′ E | Andaqueptic Haplaquoll | [45] |
| China | 121°7′ E, 31°9′ N | silt loam | [46] |

| | | | |
|---|---|---|---|
| China | 31°58′ N, 119°18′ E | Typic Haplaquepts | [47] |
| China | 26°6′ N, 119°18′ E | silt loam | [48] |
| China | 31°32′ N, 120°41′ E | Anthrosol | [49] |
| China | 31°58′ N, 118°48′ E | Irragric Anthrosols | [50] |
| China | 31°37′ N, 120°28′ E | Fluvisols | [51] |
| China | 41°32′ N, 123°23′ E | Cambisols | [51] |
| China | 47°35′ N, 133°31′ E | Phaeozems | [51] |
| China | 30°58′ N, 105°12′ E | Acrisols | [51] |
| China | 29°51′ N, 115°33′ E | Gleysol | [52] |
| China | 30°16′ N, 120°7′ E | Loam clay | [53] |
| China | 30°8′ N, 120°10′ E | Loam clay | [53] |
| China | 26°44′ N, 115°04′ E | red soil | [54] |
| China | 32°35′ N, 119°42′ E | fluvisol | [55] |
| China | 31°8′ N, 121°28′ E | clay | [56] |
| China | 31°07'–31°37′ N, 119°31′–120°03′ E | clay | [57] |
| China | 31°30′ N, 120°33′ E | Gleyic Stagnic Anthrosol | [58] |
| China | 31°33′ N, 120°43′ E | loam clay | [59] |
| China | 28°11′ N, 113°04′ E | Endogleyic Hapli-Stagnic Anthrosol | [3] |
| China | 32°35′ N, 119°42′ E | Shajiang-Aquic Cambosols | [60] |
| China | 30°53′ N, 121°23′ E | silty loam | [61] |
| China | 31°58′ N, 119°18′ E | Typic Haplaquept | [62] |
| China | 30°53′ N, 121°23′ E | silty loam | [63] |
| Philippines | 14°09′ N, 121°15′ E | Andaqueptic Haplaquoll | [64] |
| China | 29°48′ N, 106°24′ E | Cabhaplic Stagnic Anthrosols | [22] |
| Indonesia | 4°59'S, 119°34′ E | Typic Haplusterts | [24] |
| China | 31°52′ N, 118°50′ E | silt loam | [28] |
| China | 26°44′ N, 115°04′ E | Red soil | [65] |

Studies used for analysis:


1. Yang, S.; Xiao, Y. N.; Sun, X.; Ding, J.; Jiang, Z.; Xu, J., Biochar improved rice yield and mitigated $CH_4$ and $N_2O$ emissions from paddy field under controlled irrigation in the Taihu Lake Region of China. *Atmos. Environ.* **2019,** 200, 69–77.
2. Maneepitak, S.; Ullah, H.; Datta, A.; Shrestha, R. P.; Shrestha, S.; Kachenchart, B., Effects of water and rice straw management practices on water savings and greenhouse gas emissions from a double-rice paddy field in the Central Plain of Thailand. *Eur. J. Agron.* **2019,** 107, 18–29.
3. Yang, Y. D.; Xu, H. S.; Li, D. Y.; Liu, J. N.; Nie, J. W.; Zeng, Z. H., Methane emissions responding to Azolla inoculation combined with midseason aeration and N fertilization in a double-rice cropping system. *Environ. Sci. Pollut. R.* **2019,** 26, 20352–20363.
4. Zhang, G.; Ma, J.; Yang, Y.; Yu, H.; Song, K.; Dong, Y.; Lv, S.; Xu, H., Achieving low methane and nitrous oxide emissions with high economic incomes in a rice-based cropping system. *Agr. Forest Meteorol.* **2018,** 259, 95–106.
5. Berger, S.; Jang, I.; Seo, J.; Kang, H.; Gebauer, G., A record of $N_2O$ and $CH_4$ emissions and underlying soil processes of Korean rice paddies as affected by different water management practices. *Biogeochemistry* **2013,** 115, 317–332.
6. Haque, M. M.; Kim, G. W.; Kim, P. J.; Kim, S. Y., Comparison of net global warming potential between continuous flooding and midseason drainage in monsoon region paddy during rice cropping. *Field Crops Res.* **2016,** 193, 133–142.
7. Nishimura, S.; Kimiwada, K.; Yagioka, A.; Hayashi, S.; Oka, N., Effect of intermittent drainage in reduction of methane emission from paddy soils in Hokkaido, northern Japan. *Soil Sci. Plant Nutr.* **2020,** 66, 360–368.
8. Wang, Z.; Gu, D.; Beebout, S. S.; Zhang, H.; Liu, L.; Yang, J.; Zhang, J., Effect of irrigation regime on grain yield, water productivity, and methane emissions in dry direct-seeded rice grown in raised beds with wheat straw incorporation. *The Crop Journal* **2018,** 6, 495–508.
9. Liao, B.; Wu, X.; Yu, Y.; Luo, S.; Hu, R.; Lu, G., Effects of mild alternate wetting and drying irrigation and mid-season drainage on $CH_4$ and $N_2O$ emissions in rice cultivation. *Sci. Total Environ.* **2020,** 698, 134212.
10. Camargo, E. S.; Pedroso, G. M.; Minamikawa, K.; Shiratori, Y.; Bayer, C., Intercontinental comparison of greenhouse gas emissions from irrigated rice fields under feasible water management practices: Brazil and Japan. *Soil Sci. Plant Nutr.* **2018,** 64, 59–67.
11. Yang, S.; Peng, S.; Xu, J.; Luo, Y.; Li, D., Methane and nitrous oxide emissions from paddy field as affected by water-saving irrigation. *Phys. Chem. Earth, Parts A/B/C* **2012,** 53–54, 30–37.
12. Liu, S.; Zhang, L.; Jiang, J.; Chen, N.; Yang, X.; Xiong, Z.; Zou, J., Methane and nitrous oxide emissions from rice seedling nurseries under flooding and moist irrigation regimes in Southeast China. *Sci. Total Environ.* **2012,** 426, 166–171.
13. Runkle, B. R. K.; Suvočarev, K.; Reba, M. L.; Reavis, C. W.; Smith, S. F.; Chiu, Y.-L.; Fong, B., Methane Emission Reductions from the Alternate Wetting and Drying of Rice Fields Detected Using the Eddy Covariance Method. *Environ. Sci. Technol.* **2019,** 53, 671–681.
14. Ali, M. A.; Hoque, M. A.; Kim, P. J., Mitigating Global Warming Potentials of Methane and Nitrous Oxide Gases from Rice Paddies under different irrigation regimes. *Ambio* **2013,** 42, 357–368.

15. Cheng, C.; Yang, X.; Wang, J.; Luo, K.; Rasheed, A.; Zeng, Y.; Shang, Q., Mitigating net global warming potential and greenhouse gas intensity by intermittent irrigation under straw incorporation in Chinese double-rice cropping systems. *Paddy Water Environ.* **2020,** 18, 99–109.
16. Wu, X.; Wang, W.; Xie, X.; Yin, C.; Hou, H.; Yan, W.; Wang, G., Net global warming potential and greenhouse gas intensity as affected by different water management strategies in Chinese double rice-cropping systems. *Sci. Rep.* **2018,** 8, 779.
17. Tyagi, L.; Kumari, B.; Singh, S. N., Water management — A tool for methane mitigation from irrigated paddy fields. *Sci. Total Environ.* **2010,** 408, 1085–1090.
18. Balaine, N.; Carrijo, D. R.; Adviento-Borbe, M. A.; Linquist, B., Greenhouse gases from irrigated rice systems under varying severity of alternate-wetting and drying irrigation. *Soil Sci. Soc. Am. J.* **2019,** 83, 1533–1541.
19. Peyron, M.; Bertora, C.; Pelissetti, S.; Said-Pullicino, D.; Celi, L.; Miniotti, E.; Romani, M.; Sacco, D., Greenhouse gas emissions as affected by different water management practices in temperate rice paddies. *Agric., Ecosyst. Environ.* **2016,** 232, 17–28.
20. Zou, J.; Huang, Y.; Jiang, J.; Zheng, X.; Sass, R. L., A 3-year field measurement of methane and nitrous oxide emissions from rice paddies in China: Effects of water regime, crop residue, and fertilizer application. *Global Biogeochem. Cy.* **2005,** 19, GB002401.
21. Wu, X.; Wang, W.; Xie, K.; Yin, C.; Hou, H.; Xie, X., Combined effects of straw and water management on $CH_4$ emissions from rice fields. *J. Environ. Manage.* **2019,** 231, 1257–1262.
22. Qi, L.; Niu, H.-D.; Zhou, P.; Jia, R.-J.; Gao, M., Effects of biochar on the net greenhouse gas emissions under continuous flooding and water-saving irrigation conditions in paddy soils. *Sustainability* **2018,** 10, 1403.
23. Tang, J.; Wang, J.; Li, Z.; Wang, S.; Qu, Y., Effects of irrigation regime and nitrogen fertilizer management on $CH_4$, $N_2O$ and $CO_2$ emissions from saline–alkaline paddy fields in Northeast China. *Sustainability* **2018,** 10, 475.
24. Jumadi, O.; Hartono, H.; Masniawati, A.; Iriany, R. N.; Makkulawu, A. T.; Inubushi, K., Emissions of nitrous oxide and methane from rice field after granulated urea application with nitrification inhibitors and zeolite under different water managements. *Paddy Water Environ.* **2019,** 17, 715–724.
25. Sriphirom, P.; Chidthaisong, A.; Yagi, K.; Tripetchkul, S.; Towprayoon, S., Evaluation of biochar applications combined with alternate wetting and drying (AWD) water management in rice field as a methane mitigation option for farmers' adoption. *Soil Sci. Plant Nutr.* **2020,** 66, 235–246.
26. Wang, C.; Shen, J.; Tang, H.; Inubushi, K.; Guggenberger, G.; Li, Y.; Wu, J., Greenhouse gas emissions in response to straw incorporation, water management and their interaction in a paddy field in subtropical central China. *Arch. Agron. Soil Sci.* **2017,** 63, 171–184.
27. Yue, J.; Shi, Y.; Liang, W.; Wu, J.; Wang, C.; Huang, G., Methane and nitrous oxide emissions from rice field and related microorganism in black soil, northeastern China. *Nutr. Cycl. Agroecosys.* **2005,** 73, 293–301.
28. Wang, J.; Zhang, X.; Xiong, Z.; Khalil, M. A. K.; Zhao, X.; Xie, Y.; Xing, G., Methane emissions from a rice agroecosystem in South China: Effects of water regime, straw incorporation and nitrogen fertilizer. *Nutr. Cycl. Agroecosys.* **2012,** 93, 103–112.

29. Gupta, D. K.; Bhatia, A.; Kumar, A.; Das, T. K.; Jain, N.; Tomer, R.; Malyan, S. K.; Fagodiya, R. K.; Dubey, R.; Pathak, H., Mitigation of greenhouse gas emission from rice–wheat system of the Indo-Gangetic plains: Through tillage, irrigation and fertilizer management. *Agric., Ecosyst. Environ.* **2016,** 230, 1–9.
30. Oo, A. Z.; Sudo, S.; Inubushi, K.; Chellappan, U.; Yamamoto, A.; Ono, K.; Mano, M.; Hayashida, S.; Koothan, V.; Osawa, T.; Terao, Y.; Palanisamy, J.; Palanisamy, E.; Venkatachalam, R., Mitigation potential and yield-scaled global warming potential of early-season drainage from a rice paddy in Tamil Nadu, India. *Agronomy* **2018,** 8, 202.
31. Pandey, A.; Mai, V. T.; Vu, D. Q.; Bui, T. P. L.; Mai, T. L. A.; Jensen, L. S.; de Neergaard, A., Organic matter and water management strategies to reduce methane and nitrous oxide emissions from rice paddies in Vietnam. *Agric., Ecosyst. Environ.* **2014,** 196, 137–146.
32. Dong, W.; Guo, J.; Xu, L.; Song, Z.; Zhang, J.; Tang, A.; Zhang, X.; Leng, C.; Liu, Y.; Wang, L.; Wang, L.; Yu, Y.; Yang, Z.; Yu, Y.; Meng, Y.; Lai, Y., Water regime-nitrogen fertilizer incorporation interaction: Field study on methane and nitrous oxide emissions from a rice agroecosystem in Harbin, China. *J. Environ. Sci.* **2018,** 64, 289–297.
33. Nugroho, S. G.; Lumbanraja, J.; Suprapto, H.; Sunyoto; Ardjasa, W. S.; Haraguchi, H.; Kimura, M., Effect of intermittent irrigation on methane emission from an Indonesian paddy field. *Soil Sci. Plant Nutr.* **1994,** 40, 609–615.
34. Husin, Y. A.; Murdiyarso, D.; Khalil, M. A. K.; Rasmussen, R. A.; Shearer, M. J.; Sabiham, S.; Sunar, A.; Adijuwana, H., Methane flux from indonesian wetland rice: the effects of water management and rice variety. *Chemosphere* **1995,** 31, 3153–3180.
35. Adhya, T. K.; Bharati, K.; Mohanty, S. R.; Ramakrishnan, B.; Rao, V. R.; Sethunathan, N.; Wassmann, R., Methane Emission from Rice Fields at Cuttack, India. *Nutr. Cycl. Agroecosys.* **2000,** 58, 95–105.
36. Yao, Z.; Zheng, X.; Dong, H.; Wang, R.; Mei, B.; Zhu, J., A 3-year record of $N_2O$ and $CH_4$ emissions from a sandy loam paddy during rice seasons as affected by different nitrogen application rates. *Agric., Ecosyst. Environ.* **2012,** 152, 1–9.
37. Zhao, Z.; Yue, Y.; Sha, Z.; Li, C.; Deng, J.; Zhang, H.; Gao, M.; Cao, L., Assessing impacts of alternative fertilizer management practices on both nitrogen loading and greenhouse gas emissions in rice cultivation. *Atmos. Environ.* **2015,** 119, 393–401.
38. Wu, Z.; Zhang, X.; Dong, Y.; Li, B.; Xiong, Z., Biochar amendment reduced greenhouse gas intensities in the rice-wheat rotation system: six-year field observation and meta-analysis. *Agr. Forest Meteorol.* **2019,** 278, 107625.
39. Liu, X.; Zhou, J.; Chi, Z.; Zheng, J.; Li, L.; Zhang, X.; Zheng, J.; Cheng, K.; Bian, R.; Pan, G., Biochar provided limited benefits for rice yield and greenhouse gas mitigation six years following an amendment in a fertile rice paddy. *CATENA* **2019,** 179, 20–28.
40. Zhou, G.; Gao, S.; Xu, C.; Zeng, N.; Rees, R. M.; Cao, W., Co-incorporation of Chinese milk vetch (*Astragalus sinicus* L.) and rice (*Oryza sativa* L.) straw minimizes $CH_4$ emissions by changing the methanogenic and methanotrophic communities in a paddy soil. *Eur. J. Soil Sci.* **2020,** 71, 924–939.
41. Hu, Q.; Liu, T.; Jiang, S.; Cao, C.; Li, C.; Chen, B.; Liu, J., Combined effects of straw returning and chemical N fertilization on greenhouse gas emissions and yield from paddy fields in Northwest Hubei Province, China. *J. Soil Sci. Plant Nutr.* **2020,** 20, 392–406.

42. Kim, S. Y.; Gutierrez, J.; Kim, P. J., Considering winter cover crop selection as green manure to control methane emission during rice cultivation in paddy soil. *Agric., Ecosyst. Environ.* **2012,** 161, 130–136.
43. Lee, C. H.; Kim, S. Y.; Villamil, M. B.; Pramanik, P.; Hong, C. O.; Kim, P. J., Different response of silicate fertilizer having electron acceptors on methane emission in rice paddy soil under green manuring. *Biol. Fertility Soils* **2012,** 48, 435–442.
44. Zhang, A.; Cui, L.; Pan, G.; Li, L.; Hussain, Q.; Zhang, X.; Zheng, J.; Crowley, D., Effect of biochar amendment on yield and methane and nitrous oxide emissions from a rice paddy from Tai Lake plain, China. *Agric., Ecosyst. Environ.* **2010,** 139, 469–475.
45. Ku, H.-H.; Hayashi, K.; Agbisit, R.; Villegas-Pangga, G., Effect of rates and sources of nitrogen on rice yield, nitrogen efficiency, and methane emission from irrigated rice cultivation. *Archives of Agronomy and Soil Science* **2017,** 63, 1009–1022.
46. Yuan, J.; Yuan, Y.; Zhu, Y.; Cao, L., Effects of different fertilizers on methane emissions and methanogenic community structures in paddy rhizosphere soil. *Sci. Total Environ.* **2018,** 627, 770–781.
47. Fan, X.; Yu, H.; Wu, Q.; Ma, J.; Xu, H.; Yang, J.; Zhuang, Y., Effects of fertilization on microbial abundance and emissions of greenhouse gases ($CH_4$ and $N_2O$) in rice paddy fields. *Ecol. Evol.* **2016,** 6, 1054–1063.
48. Wang, W.; Sardans, J.; Wang, C.; Tong, C.; Ji, Q.; PeÑUelas, J., Effects of fertilization on porewater nutrients, greenhouse-gas emissions and rice productivity in a subtropical paddy field. *Exp. Agric.* **2018,** 55, 395–411.
49. Xia, L.; Wang, S.; Yan, X., Effects of long-term straw incorporation on the net global warming potential and the net economic benefit in a rice–wheat cropping system in China. *Agric., Ecosyst. Environ.* **2014,** 197, 118–127.
50. Xu, X.; Wu, Z.; Dong, Y.; Zhou, Z.; Xiong, Z., Effects of nitrogen and biochar amendment on soil methane concentration profiles and diffusion in a rice-wheat annual rotation system. *Sci. Rep.* **2016,** 6, 38688.
51. Xie, B.; Zheng, X.; Zhou, Z.; Gu, J.; Zhu, B.; Chen, X.; Shi, Y.; Wang, Y.; Zhao, Z.; Liu, C.; Yao, Z.; Zhu, J., Effects of nitrogen fertilizer on $CH_4$ emission from rice fields: multi-site field observations. *Plant Soil* **2010,** 326, 393–401.
52. Zhang, Z. S.; Chen, J.; Liu, T. Q.; Cao, C. G.; Li, C. F., Effects of nitrogen fertilizer sources and tillage practices on greenhouse gas emissions in paddy fields of central China. *Atmos. Environ.* **2016,** 144, 274–281.
53. Zhong, Y.; Wang, X.; Yang, J.; Zhao, X.; Ye, X., Exploring a suitable nitrogen fertilizer rate to reduce greenhouse gas emissions and ensure rice yields in paddy fields. *Sci. Total Environ.* **2016,** 565, 420–426.
54. Yuan, Y.; Dai, X.; Wang, H., Fertilization effects on $CH_4$, $N_2O$ and $CO_2$ fluxes from a subtropical double rice cropping system. *Plant Soil Environment* **2019,** 65, 189–197.
55. Yao, Z.; Zheng, X.; Wang, R.; Dong, H.; Xie, B.; Mei, B.; Zhou, Z.; Zhu, J., Greenhouse gas fluxes and NO release from a Chinese subtropical rice-winter wheat rotation system under nitrogen fertilizer management. *J. Geophys. Res.: Biogeo.* **2013,** 118, 623–638.

56. Gao, X.; Lv, A.; Wang, S.; Su, L.; Zhou, P.; An, Y., Greenhouse gas intensity and net ecosystem carbon budget following the application of green manures in rice paddies. *Nutr. Cycl. Agroecosys.* **2016,** 106, 169–183.
57. Lan, T.; Li, M.; Han, Y.; Deng, O.; Tang, X.; Luo, L.; Zeng, J.; Chen, G.; Yuan, S.; Wang, C.; Gao, X., How are annual $CH_4$, $N_2O$, and NO emissions from rice–wheat system affected by nitrogen fertilizer rate and type? *Appl. Soil Ecol.* **2020,** 150, 103469.
58. Kong, D.; Li, S.; Jin, Y.; Wu, S.; Chen, J.; Hu, T.; Wang, H.; Liu, S.; Zou, J., Linking methane emissions to methanogenic and methanotrophic communities under different fertilization strategies in rice paddies. *Geoderma* **2019,** 347, 233–243.
59. Wang, J.; Chen, Z.; Ma, Y.; Sun, L.; Xiong, Z.; Huang, Q.; Sheng, Q., Methane and nitrous oxide emissions as affected by organic–inorganic mixed fertilizer from a rice paddy in southeast China. *J. Soils Sed.* **2013,** 13, 1408–1417.
60. Xie, B.; Zhou, Z.; Mei, B.; Zheng, X.; Dong, H.; Wang, R.; Han, S.; Cui, F.; Wang, Y.; Zhu, J., Influences of free-air $CO_2$ enrichment (FACE), nitrogen fertilizer and crop residue incorporation on $CH_4$ emissions from irrigated rice fields. *Nutr. Cycl. Agroecosys.* **2012,** 93, 373–385.
61. Sun, H.; Zhou, S.; Zhang, J.; Zhang, X.; Wang, C., Year-to-year climate variability affects methane emission from paddy fields under irrigated conditions. *Environ. Sci. Pollut. R.* **2020,** 27, 14780–14789.
62. Ji, Y.; Liu, G.; Ma, J.; Zhang, G.-B.; Xu, H., Effects of urea and controlled release urea fertilizers on methane emission from paddy fields: A multi-year field study. *Pedosphere* **2014,** 24, 662–673.
63. Sun, H.; Zhou, S.; Zhang, J.; Zhang, X.; Wang, C., Effects of controlled-release fertilizer on rice grain yield, nitrogen use efficiency, and greenhouse gas emissions in a paddy field with straw incorporation. *Field Crops Res.* **2020,** 253, 107814.
64. Weller, S.; Kraus, D.; Ayag, K. R. P.; Wassmann, R.; Alberto, M. C. R.; Butterbach-Bahl, K.; Kiese, R., Methane and nitrous oxide emissions from rice and maize production in diversified rice cropping systems. *Nutr. Cycl. Agroecosys.* **2015,** 101, 37–53.
65. Liu, H.; Wu, X.; Li, Z.; Wang, Q.; Liu, D.; Liu, G., Responses of soil methanogens, methanotrophs, and methane fluxes to land-use conversion and fertilization in a hilly red soil region of southern China. *Environ. Sci. Pollut. R.* 2017, 24 8731–8743.
